# A Severe Case of Median Arcuate Ligament Syndrome with Successful Angioplasty and Stenting

**DOI:** 10.1155/2012/129870

**Published:** 2012-09-19

**Authors:** Keerati Hongsakul, Sorracha Rookkapan, Jitpreedee Sungsiri, Teeravut Tubtawee

**Affiliations:** Department of Radiology, Faculty of Medicine, Prince of Songkla University, 15 Kanchanavanit Road, Hat Yai 90110, Thailand

## Abstract

Median arcuate ligament syndrome (MAL) or celiac axis compression syndrome (CACS) is a rare etiology of chronic abdominal pain. Traditional treatment of this syndrome is surgery. We report a case of median arcuate ligament syndrome with a severe compression of the celiac trunk, which was successfully treated by angioplasty with stenting.

## 1. Introduction

**Figure 1:**
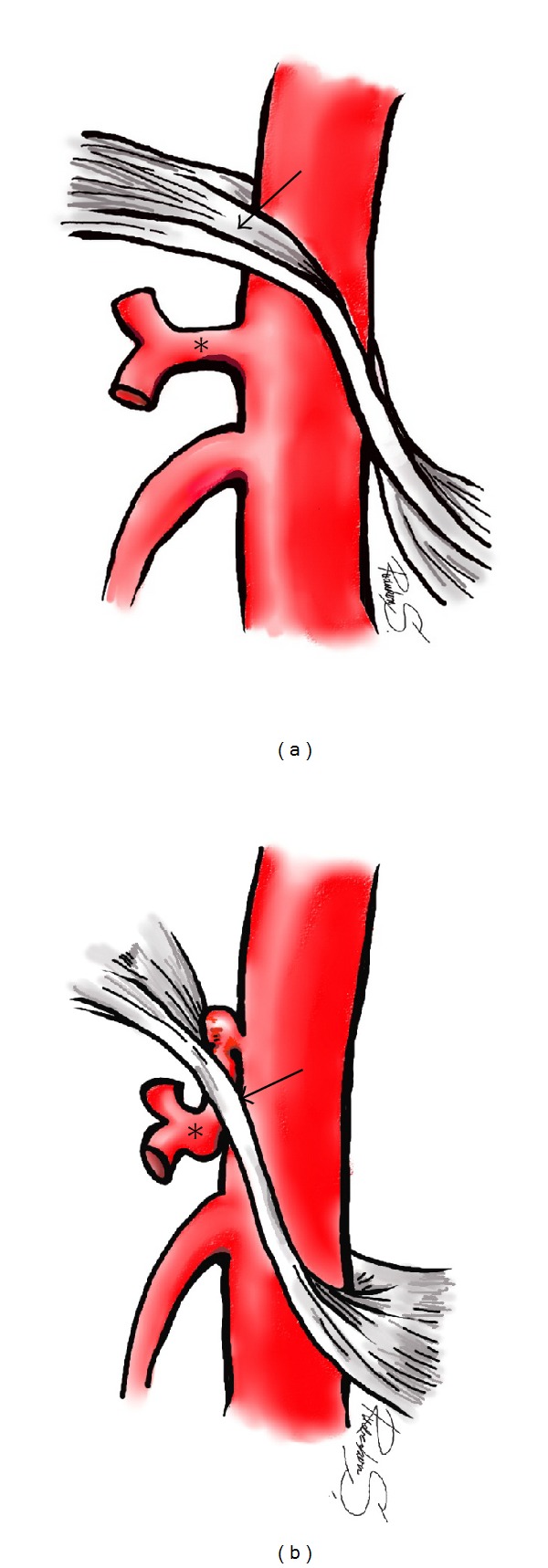
Drawing of sagittal view of normal anatomy of the median arcuate ligament (a), its normal course (arrow) passes anterior to the aorta and superior to the celiac artery (asterisk). In case of median arcuate ligament (b), the fibrous ligament (arrow) crosses proximal portion of the celiac artery (asterisk) resulting in compression.

Median arcuate ligament syndrome (MALS) or celiac axis compression syndrome (CACS) is an uncommon condition caused by the external compression of the celiac trunk by the median arcuate ligament (Figure 1). This symptom involves abdominal pain, nausea, vomiting, and weight loss. Typically, the compression of the celiac trunk is usually seen on the sagittal view of a computerized tomography scan where it appears prominent on expiration; however, in severe cases, it may be shown as a persistent compression on inspiration [1]. Traditionally, the treatment of this syndrome is surgery such as a classic opening or a novel laparoscopic division of the median arcuate ligament to relieve the extrinsic compression [2]. Percutaneous endovascular treatment is an alternative technique and may be considered in selected cases, for which the traditional surgery failed or was a treatment option. We report a median arcuate ligament syndrome case with a severe compression of the celiac trunk, presenting with chronic abdominal pain and weight loss, which was successfully treated by angioplasty with stenting. His clinical symptoms improved.

## 2. Case Presentation

**Figure 2:**
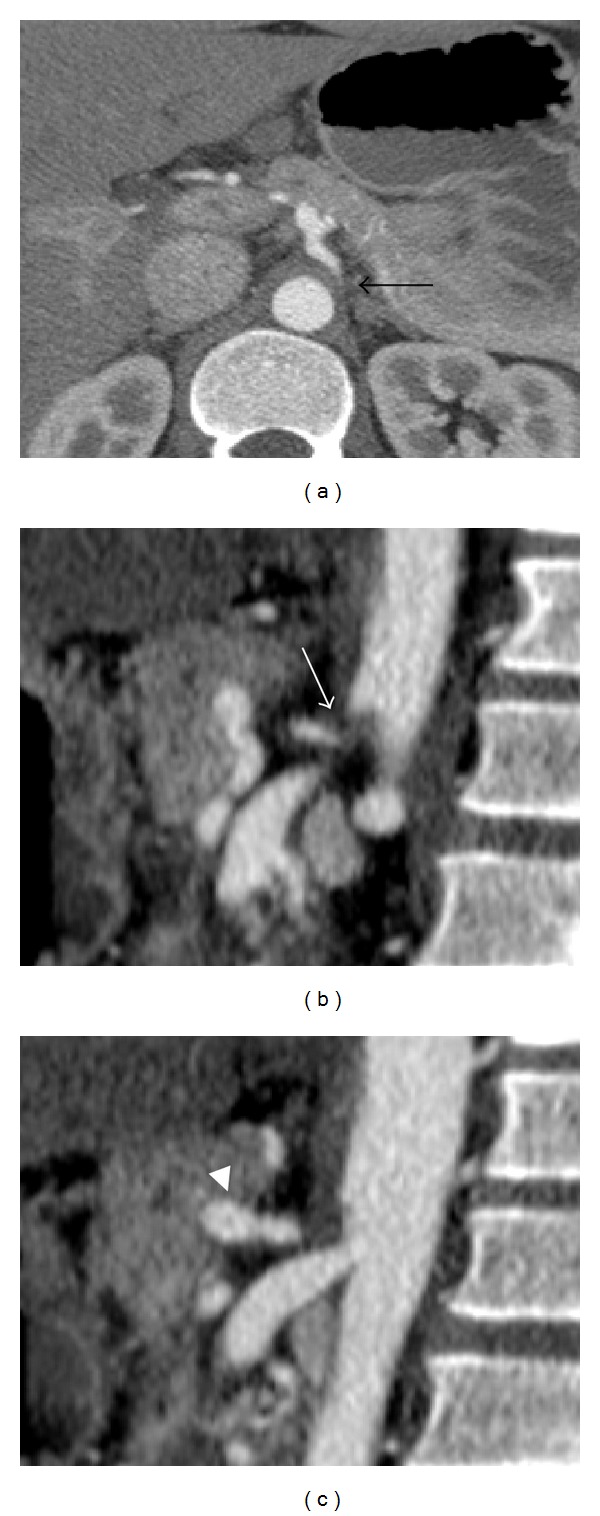
Axial (a) and sagittal (b and c) CT scans of MALS reveal severe stenosis of celiac axis from extrinsic compression (arrows) and poststenotic dilation of proximal celiac artery (arrowhead).

**Figure 3:**
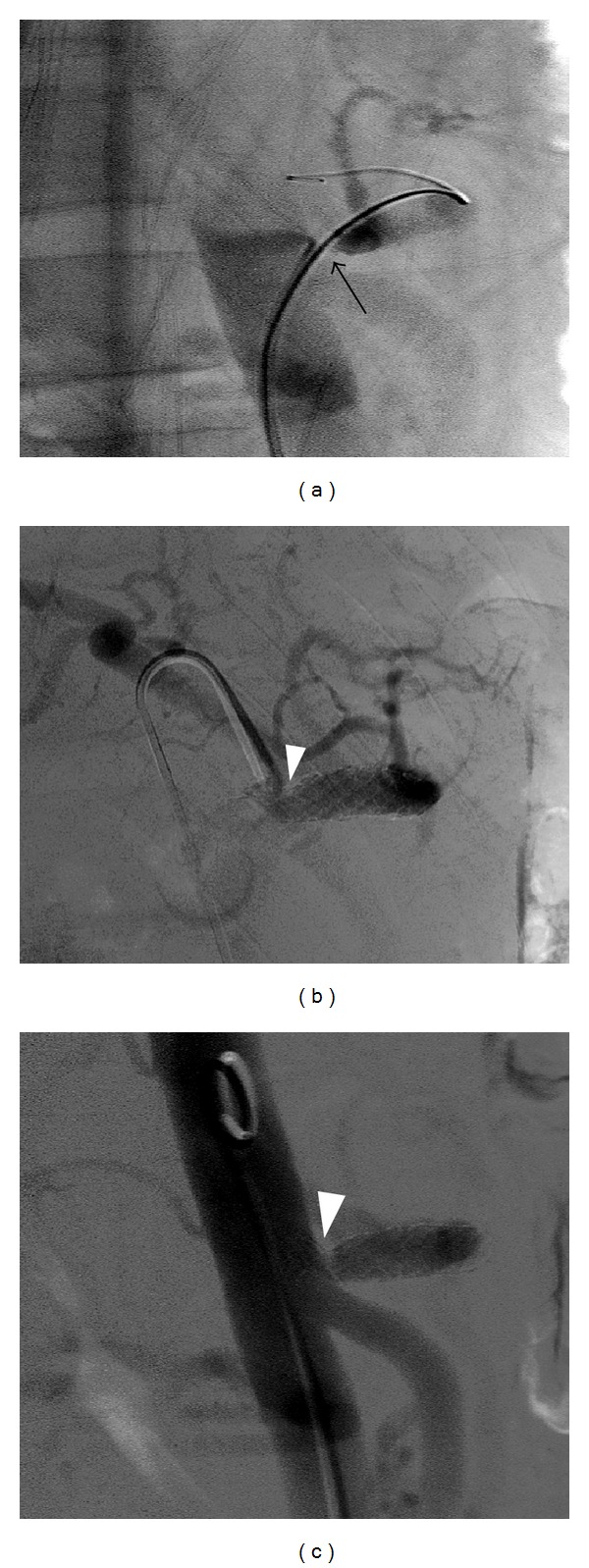
Celiac angiography of MALS (a) shows severe stenosis (arrow) with poststenotic dilation of celiac axis. Celiac angiography (b) and aortography (c) after balloon angioplasty with stenting show acceptable residual stenosis at proximal celiac artery (arrowhead).

**Figure 4:**
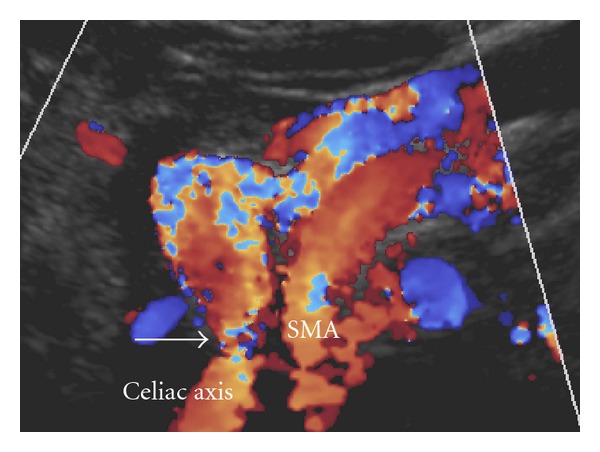
Color ultrasound study at 3-month follow up shows focal residual stenosis at proximal celiac artery.

**Figure 5:**
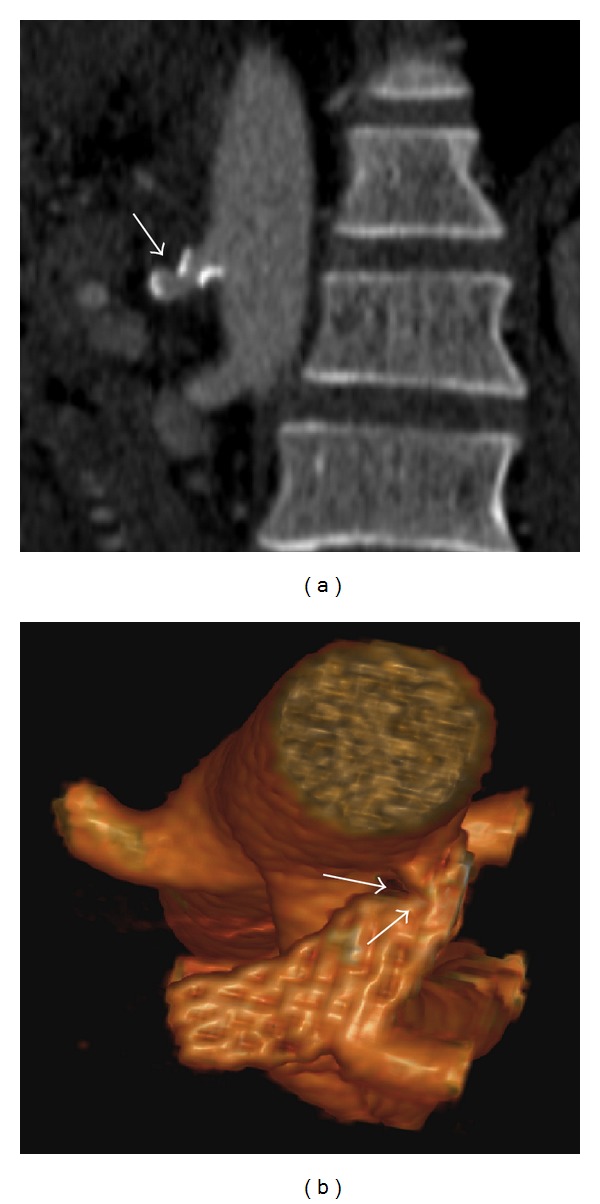
Sagittal view (a) and 3D reconstruction (b) of the CTA at 15-month follow up show stent patency and focal residual stenosis (arrows) at proximal celiac artery.

A 27-year-old male, who had not any past medical history, presented with chronic intermittent abdominal pain for 10 months, during which he had experienced a 20-pound weight loss. His whole abdomen sonogram, esophagogastroscopy, and colonoscopy were within normal limits. However, the computer tomography (CT) of the abdomen revealed a severe compression of celiac axis (Figure 2). According to his clinical symptoms and imaging findings, the patient was diagnosed with MALS. The patient was treated by exploratory laparotomy involving the releasing of the medial arcuate ligament. However, his symptom did not significantly improve. His follow-up CT angiography (CTA) at 1 month showed persistent stenosis of the celiac axis, so he was referred to the Interventional Radiology unit for endovascular treatment. The procedure started with aortogram, using 5 French size of pigtail angiographic catheter (Boston Scientific, France). It showed severe stenosis at proximal portion of celiac trunk associated with enlarged superior mesenteric artery and multiple collateral arteries. Then, the selective celiac angiogram was performed using 5 French size of Chuang-C angiographic catheter (Boston Scientific, France). The celiac angiogram confirmed focal severe (95%) stenosis at the proximal portion (Figure 3(a)). The endovascular treatment involved the predilatation of the stenotic site using a 4 × 20 mm balloon (PowerFlex, Cordis, the Netherlands) followed by the deployment of a 8 × 40 mm bare metal stent (Palmaz-Genesis, Cordis, the Netherlands) at 8 atmospheres and postdilatation at the same pressure. The final angiographic results revealed an improved stenosis (30% residual stenosis) (Figures 3(b) and 3(c)). The color ultrasound at the 3-month follow up showed that the stent was patent (Figure 4). The patient was discharged on the second postoperative day with minimal residual abdominal pain. At the 15-month follow up, the patient was symptom-free and regained his lost weight. His CTA revealed a patent celiac stent with an unchanged degree of residual stenosis due to external compression (Figures 5(a) and 5(b)).

## 3. Discussion

The MALS or CACS is a rare cause of postprandial pain and weight loss. This syndrome was first described by Harjola [3] in 1963. The incidence of MALS has been found in 10–24% [1]. The etiology is celiac artery compression by the medial arcuate ligament (MAL) resulting in compromised blood flow and symptom causation [1–4]. However, some patients are asymptomatic due to sufficient collateral supply from superior mesenteric circulation. This syndrome is more frequent among young females with a thin body habitus and comprises the classical triad: postprandial abdominal pain, epigastric bruit, and the presence of extrinsic celiac compression revealed by vascular imaging [5]. 

The goal of MALS treatment is restoring normal blood flow in the celiac axis [2]. Classically, a simple surgical division of the fibrous ligament was performed. Other complex surgical procedures like vascular reconstruction of the celiac artery with patch angioplasty, aortoceliac bypass, and reimplantation of the celiac artery may be needed in some patients. However, open surgery is more invasive and increases the morbidity rate. Previous studies have reported a clinical improvement after operation ranging between 65% and 80% at the 1–18 year follow up time period [2, 6]. The recently application of laparoscopy in the division of MAL has proven to be a novel technique because it is less invasive and involved with a lower morbidity rate than open surgery [7]; yet, the results from both procedures are equal. However, the outcomes from several of these studies are based on only short follow up periods [7]. 

Percutaneous angioplasty with stenting is an alternative technique for the treatment of MALS. It is a minimally invasive technique, characterized by short hospitalization and low morbidity rate. However, there are a few studies that have reported successful of endovascular treatment. One of them is the study by Silva et al. [8], in which stenting was employed in four patients with extrinsic compression of the celiac artery with immediate excellent results. Only one of the four patients had a 3-year symptom-free follow up period. However, a long term of prognosis of the angioplasty and stenting is still unknown because a few treated cases were reported. In our opinion, endovascular treatment is beneficial in some selected cases such as those with a failure of traditional surgery, as in the case presented here, or contraindication to surgery. Moreover, cases of permanent changes in the celiac artery with inadequate flow after successful surgery may benefit from adjunct intraluminal treatment. 

In summary, percutaneous angioplasty with stenting is an alternative technique for the treatment of selected cases of MALS cases with treatment failure by or contraindication to traditional surgery and may be employed as additional treatment after surgery.
